# Indoor Navigation by People with Visual Impairment Using a Digital Sign System

**DOI:** 10.1371/journal.pone.0076783

**Published:** 2013-10-08

**Authors:** Gordon E. Legge, Paul J. Beckmann, Bosco S. Tjan, Gary Havey, Kevin Kramer, David Rolkosky, Rachel Gage, Muzi Chen, Sravan Puchakayala, Aravindhan Rangarajan

**Affiliations:** 1 Minnesota laboratory for Low-Vision Research, University of Minnesota, Twin Cities, Minnesota, United States of America; 2 University of St. Thomas, St. Paul, Minnesota, United States of America; 3 University of Southern California, Los Angeles, California, United States of America; 4 Advanced Medical Electronics Corporation (AME), Maple Grove, Minnesota, United States of America; Harvard Medical School, United States of America

## Abstract

There is a need for adaptive technology to enhance indoor wayfinding by visually-impaired people. To address this need, we have developed and tested a Digital Sign System. The hardware and software consist of digitally-encoded signs widely distributed throughout a building, a handheld sign-reader based on an infrared camera, image-processing software, and a talking digital map running on a mobile device. Four groups of subjects—blind, low vision, blindfolded sighted, and normally sighted controls—were evaluated on three navigation tasks. The results demonstrate that the technology can be used reliably in retrieving information from the signs during active mobility, in finding nearby points of interest, and following routes in a building from a starting location to a destination. The visually impaired subjects accurately and independently completed the navigation tasks, but took substantially longer than normally sighted controls. This fully functional prototype system demonstrates the feasibility of technology enabling independent indoor navigation by people with visual impairment.

## Introduction

Independent mobility is an important prerequisite for full participation in modern society. People who are visually impaired often experience reduced mobility affecting vocational, educational, and recreational opportunities, and activities of daily life such as shopping and access to health care. Reduced mobility may also result in social isolation and depression [1].

Two major aspects of pedestrian mobility are obstacle avoidance and spatial navigation. Obstacle avoidance refers to the ability to take the next step safely without bumping into things, or tripping. Many people who are visually disabled deal effectively with obstacle avoidance using a white cane, dog guide, or their own low vision. Spatial navigation, sometimes termed “Wayfinding,” refers to the ability to learn layouts, and plan and follow routes from place to place while correctly updating current location and heading.

Compared with obstacle avoidance, much less is known about wayfinding with vision impairment, and there is no technology equivalent to the success of the white cane or dog guide. GPS technology has been exploited for speech-based navigation for visually-impaired wayfinding outdoors (see [2] for an evaluation of four commercially available systems). But indoors, away from windows, GPS signals are not reliable, nor do they provide adequate spatial resolution for finding rooms and other key locations. While several location systems are feasible for indoor applications [3,4], none has seen any significant adoption. Their limitations are due to cost and other difficulties Including installation and maintenance of active sensors, or reliability of devices relying on detection of features in un-instrumented environments (e.g. using computer vision or detection of existing WI-FI signals). Location sensing for visually impaired users poses additional challenges. To be useful, the system has to be reliable since verification and error detection by blind users is more difficult. This rules out most of the visual-scene analysis and unassisted dead-reckoning techniques. Because the user population is relatively small, systems relying on installing and maintaining a vast array of sensors or beacons, or those requiring extensive and purpose-specific mapping efforts are hard to justify on economic grounds. Systems using fiducial and informational markers require only minor change to the infrastructure, and have seen some level of commercial application [5]; however, such systems require a user to visually locate the markers and aim a camera at them. One approach for adapting such a system for blind users is to make the markers salient to a machine vision system. Coughlan and Manduchi took this route with colorful markers that can be easily detected with the camera on a mobile phone [6,7]. We took a similar approach but in the infrared range to avoid using conspicuous markers that could adversely affect the aesthetics of the environment [8].

A recently published survey of electronic travel aids for the blind [9] identified nine commercially available navigational aids, but none were designed for indoor usage. An interesting new system from In Touch Graphics allows blind pedestrians to download indoor or outdoor directions in text or auditory formats, or obtain the equivalent verbal routes by phone (www.ClickAndGoMaps.com). Additional systems under development make use of RFID tags [10-12], WI-FI triangulation [13], ultrasound triangulation [14], laser scanning [15] and computer vision [16]. Manduchi and Coughlan [17] provide a useful review of the limitations to date and future potential of computer vision for visually-impaired wayfinding.

Havik et al [10] tested blind and low-vision subjects in indoor route-following tasks similar to our Experiment 3, using a navigation system based on RFID tags. Similar to our results reported below, they found that visually impaired subjects responded positively to the information provided by their system about route following and nearby landmarks. Arditi and Tian [18] surveyed a group of ten experienced blind travelers regarding their preferences for a camera-based wayfinding device. Consistent with our design, the blind travelers placed a high priority on finding architectural features such as doorways and stairs.

There are three key components of a successful indoor technology for wayfinding. First, location sensing determines the person’s current location in the map and their orientation. Second, a digital map of the space should represent spatial information in a data structure appropriate for conveying navigation-relevant messages about surroundings and routes to a user. Such information is not explicitly represented in typical file formats (DWG, REVIT) used for architectural drawings. Some systems supporting indoor navigation rely on fixed messages--Talking Signs (www.talkingsigns.com), and Talking Lights (www.talking-lights.com)-- without incorporating an explicit map of the space. Finally, the interface must convey spatial information to a visually-impaired user in a non-visual format. While various auditory and tactile interface designs have been studied and implemented (for a review, see [19]), computer speech is the most widely available and economical output modality and was preferred by the subjects surveyed by Arditi and Tian [18].

We have developed technology for non-visual indoor navigation incorporating these three main features. We call it the *Digital Sign System* (DSS). In this paper, we will summarize the hardware and software features of DSS and present results from human performance testing with blind and low-vision participants.

**Figure 1:**
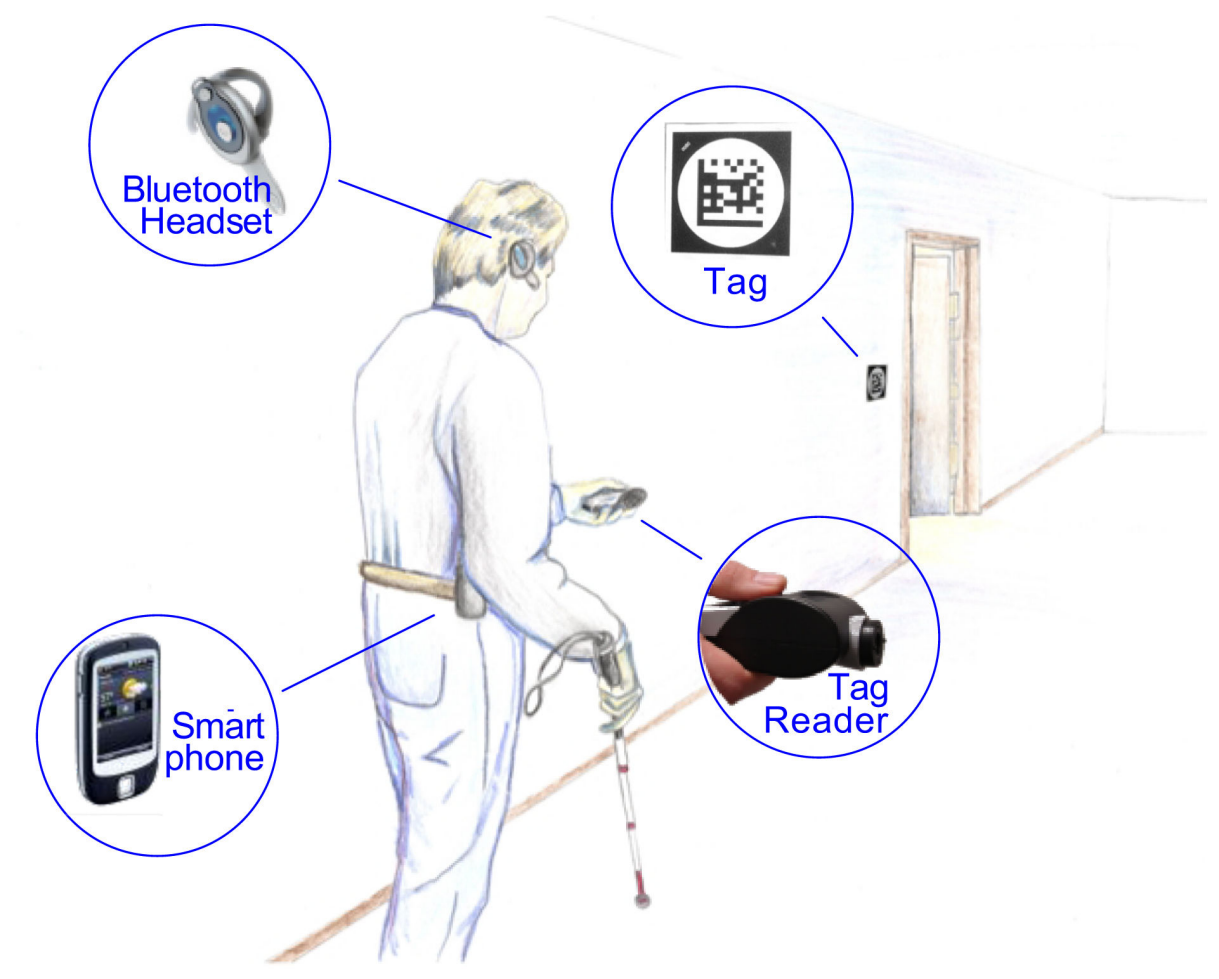
Schematic drawing of the Digital Sign System (DSS).

Figure 1 presents a schematic overview of DSS.

The details of DSS will be described in the next section. As an overview, DSS makes use of 2-D matrix barcoded signs posted at room entrances or other key locations within a building. Since some of the DSS bar codes have roles different from conventional signage (e.g., as position beacons), we often refer to the bar codes by the generic term “tag” rather than “sign”. The pedestrian uses a hand-held reader to detect the digital tags. The reader illuminates the tags in the environment with infrared light and uses an infrared camera to detect the tags and recover a unique number from each tag. The number identifies a location in the building. Software running on a mobile device contains a digital map of the building. The mobile device uses synthetic speech to communicate information to the pedestrian about the current location or routing instructions to a destination. The software on the mobile device is called *Building Navigator* and integrates information from the digital tags and the digital map in response to user inputs.

## DSS System Description

### Digital Signs

**Figure 2:**
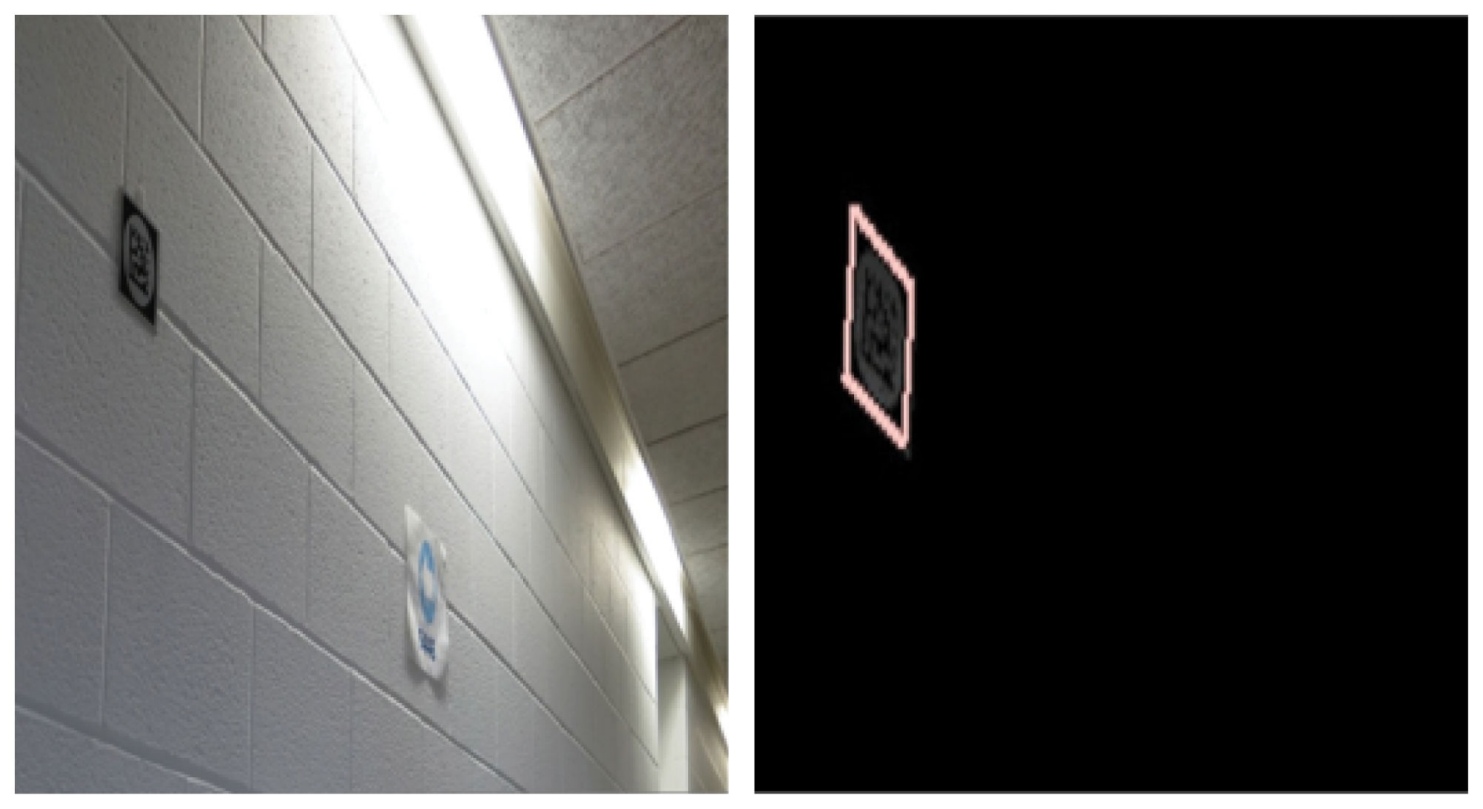
Segmentation of a Digital Tag’s image from its background. Left: a digital tag on a wall. Right: the IR image of the same tag demonstrating the segmentation advantage offered by the retro-reflective effect and unique patterns within the tag design.

The digital signs (tags) are based on Data Matrix 2-D bar codes. Each tag encodes a 16-bit hexadecimal number. To provide robust segmentation of tags from the surroundings, the 2D matrix is imbedded in a unique circle-and-square background. The tags used in our human testing measured 10 cm square and were consistently mounted between 1.5 and 2 meters from the floor on the wall, typically next to doors, within long stretches of walls without doors, and at key points in intersections. To enhance the salience of the tags for image capture, they were printed on 3M infrared (IR) retro-reflective material. When the tag was illuminated by an infrared beam from the user’s tag reader, the IR reflections yielded a very bright tag image against almost any cluttered background. An example is shown in Figure 2. Notice how the bright lights at the top of the wall and unrelated paper signage disappear in the IR image. The resulting high signal-to-noise ratio and the unique pattern within the tag design provide reliable segmentation of the tags from the clutter and virtually eliminate false positive detections

**Figure 3:**
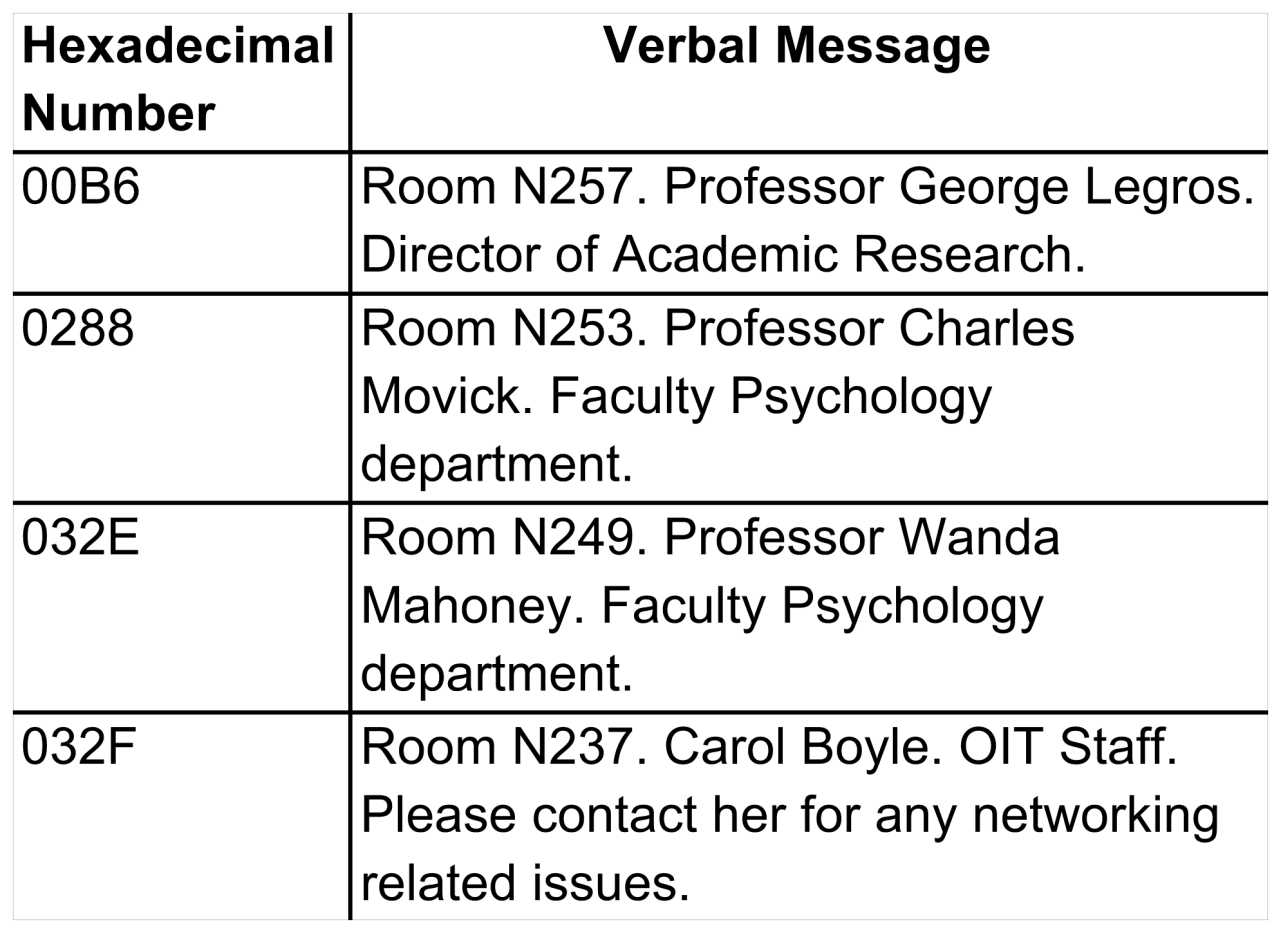
Example of a look-up table used in the Tag Browsing Mode. The table contains hexadecimal sign numbers and corresponding text messages.

The DSS system can operate without a map of the floor plan. As shown in Figure 3, the software can simply refer to a look-up table which associates hexadecimal tag numbers with a verbal message. We refer to this mode of operation as “tag browsing”. When the user’s tag reader recovers the numerical value from a sign, the synthetic speech interface announces the corresponding text description from the look-up table. Tag browsing provides the user with fixed messages, analogous to Talking Signs or Talking Lights, but not map-based information about arbitrary routes or layout

### Tag Reader

The current version of the tag reader is a major revision of our earlier design [8]. The tag reader consists of a camera, lens, IR illuminator, computer-on-module (COM), custom printed circuit boards, a Bluetooth radio, a standard 12 key keypad, rechargeable batteries, and a housing. The camera is 1.3 mega-pixel (1280 x 960) monochrome Flea 2 USB 2.0 camera from Point Grey Research. The COM is a CoreExpress-ECO from Lippert. We designed two custom printed circuit boards. The first mates with the COM and implements I/O, power management, and a keypad interface. The second mates to the first board and drives the IR illuminator, which is a ring containing 4 IR LEDs. We purchased a commercial off-the-shelf lens and an IR bandgap filter as well as a Bluetooth USB radio, keyboard, and rechargeable batteries. We designed a custom housing.

The COM hosts the Embedded Windows XP operating system. We developed an image capture and processing application in the C++ programming language. This application captures an image from the USB camera and performs the following image processing functions: Canny edge detection, segmentation, shape identification, and data matrix decoding. The image-processing algorithm is capable of detecting ellipses and quadrilaterals at high speed, which is needed to detect the 2D tags’ circle and square background when the tag is “viewed” from a general viewpoint. If it detects a quadrilateral that circumscribes an ellipse (and the quadrilateral’s centroid is near the midpoint of the ellipse’s foci), the application normalizes and decodes the tag. The application processes approximately 10 frames per second and wirelessly transmits tag information to the Building Navigator via Bluetooth.

The field of view (FoV) of the tag reader is 35° horizontal by 22° vertical (measured empirically by moving tags through the field of view of the reader).

**Figure 4:**
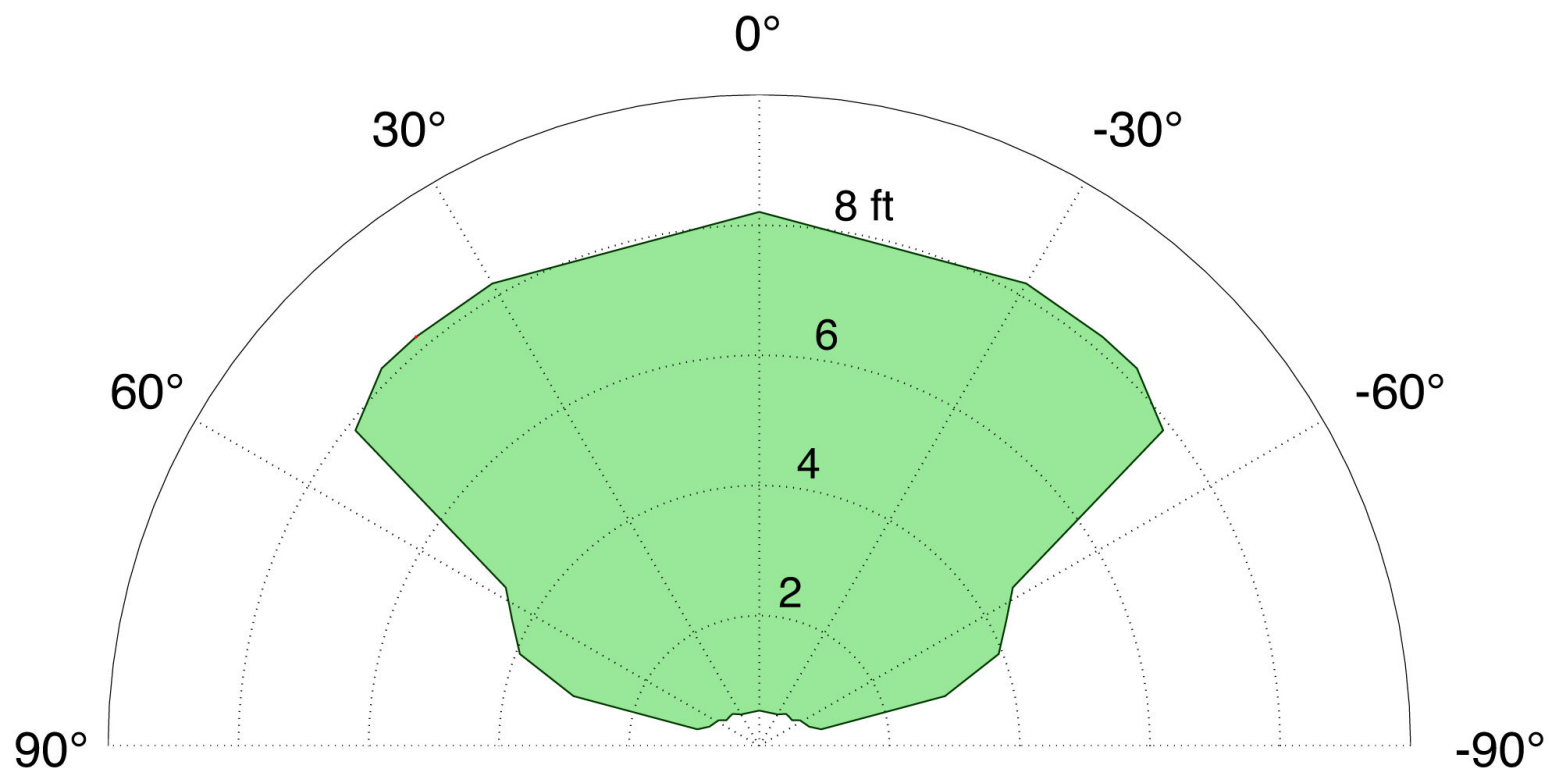
Operating range of the Tag Reader (green). The boundaries of the green region show the maximum and minimum distances for successful reading of the tags as a function of the angle between the tag reader and the normal to the tag.

Figure 4 shows the operating range of the tag reader. The outer boundary of the green region shows the maximum distance for reading the tag as a function of the angle between the tag reader and the normal to the tag. The reader can reliably identify the coded numbers on the tags out to about 8 ft and for angles of incidence up to about 55°. The upper bound on distance is determined by the resolution of the infrared camera and the sizes of the features on the digital tags. The range could be extended with a higher resolution camera or larger digital tags. The angular limit is due to the foreshortening of the tags in the image and the properties of the retro-reflective material. The inner boundary of the green region in Figure 4 shows that tags can be read successfully at distances under one foot for angles of incidence within 60°. This moderately large operating range means that a user can walk along a corridor while easily monitoring the wall-mounted tags. Once a tag is detected and announced by the interface, the orientation of the tag reader (e.g., pointed left or right) provides the user with information about the direction of the room or other feature. In our case, tags announcing room numbers were usually mounted at a standard location to the left of the room door. In most cases, this arrangement made it easy for our subjects to find a particular door.

### Digital Maps

The navigable portion of the floor plan of a building consists of interconnected 2D spaces separated by walls. The primary types of spaces are rooms, lobbies, hallways and intersections. In addition, local features must be represented, such as tags, doors, elevator thresholds, and staircase entrances. We designed digital maps for the DSS to capture these types of information. A scaled architectural floor plan was first reduced to its walls and the floor space within the building’s footprint. This floor space was tessellated with triangles, called *base polygons*, such that no triangular edge crossed a wall. Semantic entities, termed overlay polygons, such as hallways, intersections, lobbies, rooms, elevators, staircases and other elements of the floor plan were built up from contiguous sets of base polygons. Overlay polygons represent spatial entities that are directly relevant for navigation. Tags were represented by points in a floor plan, and were associated with an edge of a particular base polygon. Routes through the space were computed in terms of adjacent overlay polygons.

Each navigational feature (an overlay polygon) is associated with one or more textual descriptions that can be spoken to the DSS user. The *fixed description* is supplied at the time the overlay polygon is created and is the default description used when that entity is queried. The fixed description only requires information from the architectural floor plan. For example, “Room N234” would be a typical fixed description of a room. Optional text descriptions will be explained in connection with Experiment 2 below.

The spatial and textual information was stored in an SQL database. Files containing SQL queries (commands) with embedded data were used as the intermediary format for transfer of databases between the maintenance programs, a web-based server, and the internet-connected tablet computer running Building Navigator.

### User Interface

We have tested DSS with the Building Navigator software running on a Windows XP "smart phone" and also a Windows handheld tablet computer. In both cases, the user interacts with the system via a standard 12-button phone keypad for input (10 digits, # and * symbols), and synthetic speech output. User-accessible functions were available in hierarchically organized menus. The user pressed ‘2’ and ‘8’ keys to move up and down through the menus. All of our blind and low-vision subjects had previous experience with voice menu systems for technology access, and had little trouble adapting to the interface.

The Building Navigator software allows three modes of operation termed Tag Browsing, Exploration, and Route Following. These three modes will be described in the description of human performance testing below.

### Using the Digital Map and Interface Without the Tags and Reader

The Building Navigator software can be useful, even in the absence of the digital tags and reader. In this case, the Tag Browsing functionality is lost. But, if the user can determine his/her current position by other means, such as reading a tactile sign, the software can provide helpful information in both the Exploration and Route Following modes. In this case, the software acts as a talking map of the building, without the location sensing capability. Our human performance testing includes tasks in which this “talking map” functionality was tested.

It should also be noted that the Building Navigator software could be used in conjunction with other forms of location sensing. In fact, we used an earlier version of Building Navigator in conjunction with location sensing based on the relative intensity of Wi-Fi signals from existing 802.11 access points in our building using a variant of the RADAR algorithm [20]. Ultimately, we were not successful in using this Wi-Fi fingerprinting method of localization because of the sparse distribution of the hubs and instability of their signal intensity.

## Human Performance Testing

We conducted three experiments with human subjects, to be described in the subsections on Experiments 1, 2 and 3 below. Our four general goals were:

- To test the reliability and functionality of the integrated system of digital tags, tag reader, digital maps, and human interface.
- To determine if participants could use the software and hardware to accomplish the intended functions of the system.
- To assess the accuracy and speed of the participants’ independent use of DSS for indoor navigation.
- To compare the performance of four user groups (see below).

### General Methods

#### Subjects

Many subjects took part in usability testing over a period of years during incremental development of DSS. The present results are based on data collected using the current version of DSS. There were four groups of subjects: 1) Five sighted controls (1 Male and 4 female, mean age 22.2 years and range 21-24) performed the exploration and route-finding tasks without the aid of DSS, using standard building signage and their normal visually-guided navigation skills. The purpose of the control group was to provide a baseline estimate of the time to complete the navigation tasks. 2) Ten blindfolded normally sighted subjects (all female, mean age 21.7 years and range 19-26) performed the tasks in the three experiments non-visually. They initiated movement through the building in response to information provided by the speech interface, but were guided by a sighted experimenter to avoid obstacles. This group represents the performance of young adults with little non-visual mobility experience, perhaps similar to recently blinded subjects who have not had mobility training. Of course, these subjects, for whom nonvisual mobility was temporary, would not experience the same emotional reactions as people with recently acquired permanent blindness.

3) Ten blind subjects (4 male and 6 female, mean age 51.6 years and range 24-70) performed the tasks in the three experiments independently, aided only by warnings from a sighted experimenter about impending obstacles. Five of the blind subjects used a dog guide, three used a white cane, and two chose to do the tasks without an aid. 4) Finally, ten low-vision subjects (3 male and 7 female, mean age 41.6 years and range 21-69) also performed the three experiments. Four of them used a white cane and six relied on their vision for obstacle avoidance.

The blind and low-vision subjects were selected based on their self-reported ability to navigate independently and to be healthy enough to undergo at least three hours of training and testing involving mobility tasks. For purposes of this study, “low vision” refers to subjects who had some useful vision for detecting large architectural features such as doorways and intersections, but insufficient vision to easily use building signage. “Blind” subjects were those who relied entirely on non-visual cues for navigation.

For practical recruiting reasons, we did not attempt to match our groups for age and gender. All of our groups had more females than males, and our two visually-impaired groups were older than our blindfolded sighted group and sighted-control group. It is possible that age and gender interacted with our group conditions to affect our data. For instance, it is known that both age per se and gender have an impact on preferred walking speed for healthy, normally sighted adults. But the effects on preferred walking speed are relatively small--less than 5% difference for females in their 20s to 50’s [21].

All subjects reviewed and signed a consent form; it was read to subjects with visual impairment. The research procedures and informed consent were approved by the University of Minnesota’s IRB. Subjects were paid for their time.

#### Procedure

Most subjects completed testing in one long session lasting three to four hours. Approximately 1.5 hours was spent training subjects with the DSS hardware and software, and introducing them to its functions. A lengthy stretch of corridor, including an L-junction, near our laboratory was fitted with digital tags and used for training. The general architectural characteristics of this training space and the placement of tags were similar to the region of the building used for data collection, but differed in corridor layout and room numbering. Subjects were shown the tags and instructed how to hold and aim the tag reader in order to “read” the tags. They were shown the three functions to be tested, and given opportunities for practice with each. They learned the corresponding interface commands.

The DSS speech interface tells distance information to users in standard units (feet or meters) and also in number of steps. In a previous study, we learned that variability in step length is low for both visually impaired and sighted pedestrians implying that number of steps can be an accurate metric for describing distance in pedestrian navigation [22]. We also found that visually impaired subjects performed better in route-following tasks when distance was conveyed by number of steps rather than in feet or in travel time [23]. To use number of steps as a distance metric, it is necessary to take into account individual differences in mean step length. To perform this calibration, we measured the distance covered by our subjects in four 10-step walks during the training procedure. The resulting mean step length was entered into the Building Navigator software as a setting. Most, but not all, of our subjects chose to use the step-count information while performing the experiments.

Following training, subjects were taken to an unfamiliar floor of our building for testing. Although a few subjects in each of the groups had visited the building before, we don’t believe any of them had a detailed mental map of the corridor structure and room numbers on the test floor. The floor was populated with digital tags next to every door, with additional tags at intersections and along blank sections of hallway without doors, all mounted on the corridor wall at a standard height of 5 ft. Prior to testing, none of the subjects were permitted to preview the distribution of tags (the blindfolded sighted group had their blindfolds in place when they exited the elevator onto the test floor.) Most of the doors in the building also had braille and embossed-letter signs that could be read by touch. The subjects were all tested in the same order in the three experiments—tag browsing (Exp. 1), exploration (Exp. 2), and route finding (Exp. 3). Testing typically happened during normal business hours, and had to contend with routine pedestrian traffic, sounds from open office doors, and other indoor environmental sounds.

Following the three experiments, the subjects filled out a short questionnaire asking for their opinions about DSS. We will report on their responses after presentation of the experimental results.

The experimental data obtained in this study are available upon request from the corresponding author.

### Experiment 1. Tag Browsing

How reliably can people find the tags non-visually with the tag reader? To address this question, participants walked up and down through a long 116 ft corridor four times (twice in each direction). There were nine tags on one side of the corridor and 10 on the other. They were instructed to walk at a comfortable pace without stopping, and to use the tag reader to identify all the digital tags on one side of the corridor. When a tag was successfully recognized, the speech interface reported the room number aloud. In total, there were 38 possible tag-recognition events in the four traversals of the corridor. Accuracy was measured as the percentage of successful recognition events out of a total of 38.

**Figure 5:**
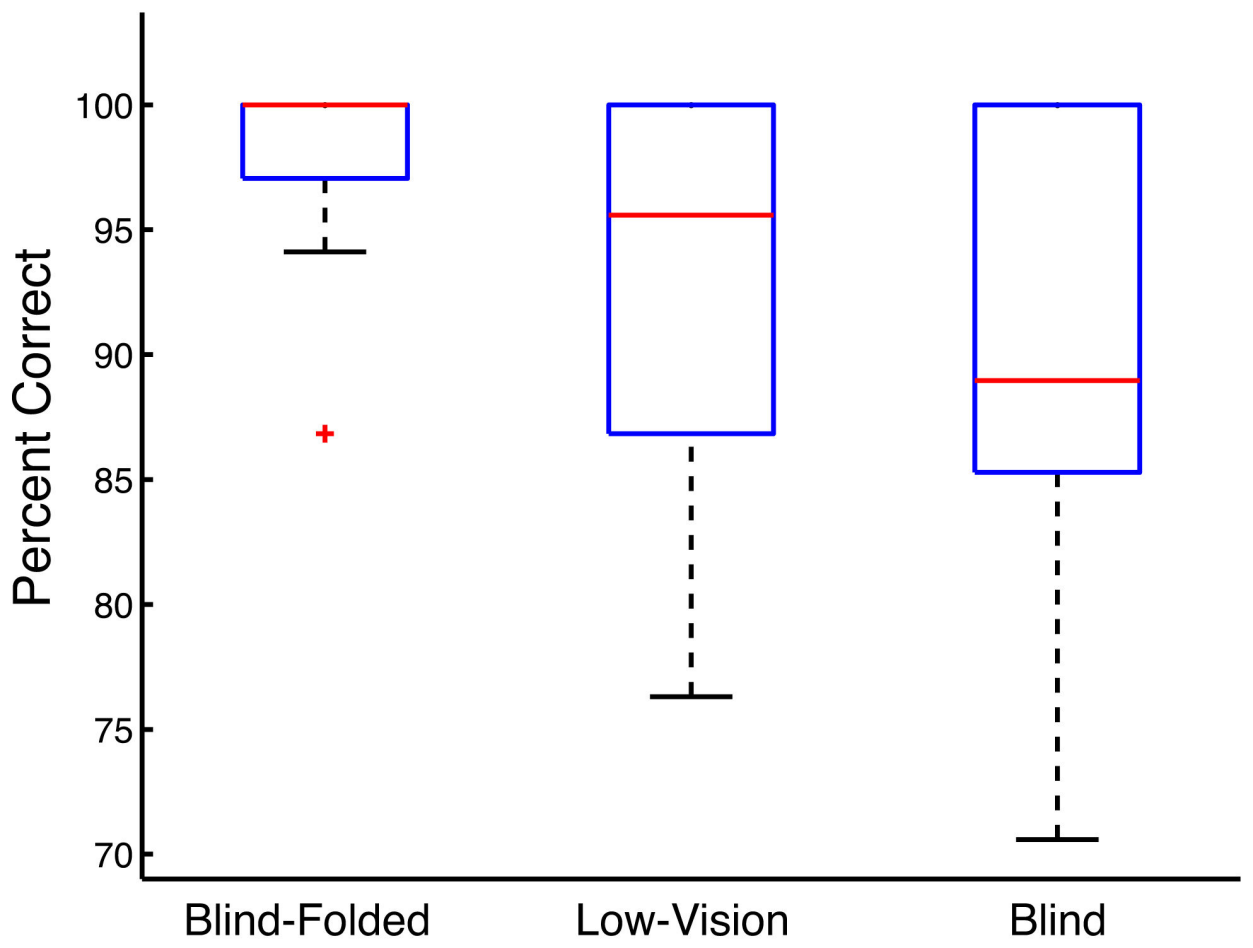
Tag Browsing Performance. Accuracy (the percentage of successful tag-recognition events out of a total of 38 opportunities during four traversals of a corridor) in the Tag Browsing Experiment: Medians (red lines), the 25% and 75% quartile limits (blue boxes), and the minimum and maximum values (whiskers) are shown for three groups of subjects.

Figure 5 shows median accuracy and 25%- and 75%-quartile limits for the three groups. The subjects in all three groups were able to do the task quite easily, with overall median accuracy of 97.4%. Although the Blind group had the lowest accuracy (median = 89%) and greatest variability (range 71% to 100%), a Kruskal-Wallace test showed no significant group differences (p = .096).

We conclude from Experiment 1 that the DSS hardware and software can be used with high accuracy by visually impaired pedestrians to read digital tags during normal, indoor corridor mobility.

### Experiment *2*: Exploration

**Figure 6:**
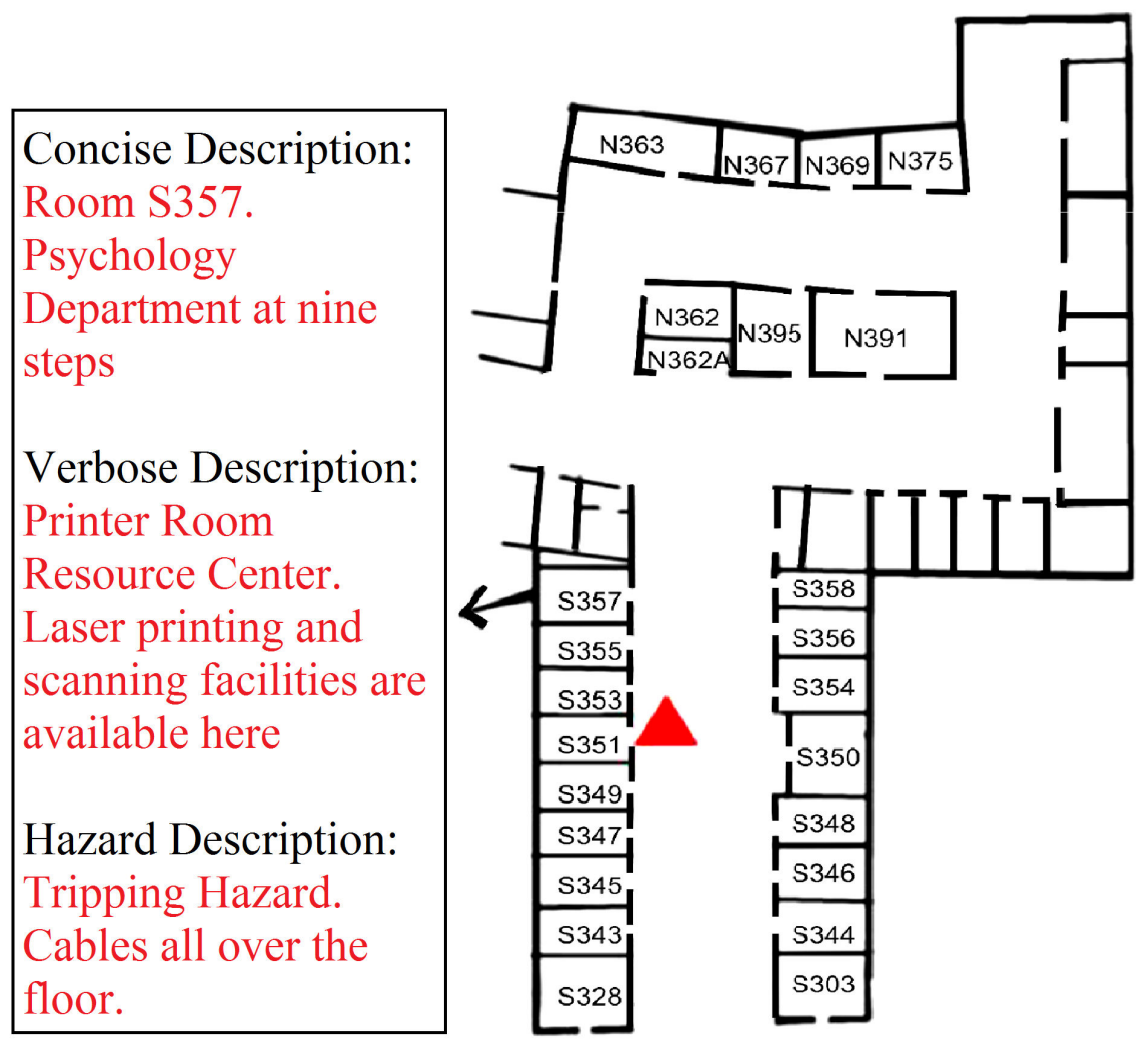
Building Navigator Explore Mode. A subject’s location and north-facing direction are indicated by the red triangle in the floor plan. See the text for a description of the functionality of the Explore mode.

Figure 6 illustrates DSS’s exploration mode. The figure shows a portion of a floor plan.

The red triangle shows a user’s current location and facing direction in a long north-south corridor. In the Exploration mode, the pedestrian uses buttons on the keypad for information about rooms ahead on the left or right, or behind on the left or right. For instance, pressing the “3” key generates a list of the three rooms ahead on the right between the pedestrian and the next intersection. For any given room, there may be up to 3 levels of description as illustrated in the figure for Room S357. The Concise Description contains the room number, possibly supplemented by a title descriptor such as the occupant’s name or the department’s name. An optional Verbose description (signaled by an auditory icon) can contain a text field of arbitrary length. A different auditory icon alerts the user about a Hazard description (if any).

We tested the Exploration mode by placing subjects at a starting location in an unfamiliar corridor and asked them to use the system to find another room on the same corridor. We timed how long it took them to reach the target room. Each subject did four trials with and without the tag reader in counterbalanced order. Subjects used the interface commands to locate the direction and distance of the target room, and then proceeded to walk to the target. When the tag reader was available they could use it to monitor the rooms they passed and to verify arrival at the target room. When they performed the task without the tag reader, they were asked to verify arrival at the target room by using touch to read the adjacent braille/embossed signs. The sighted control group simply used vision to find the target room and walk to it; they did not use DSS technology.

**Figure 7:**
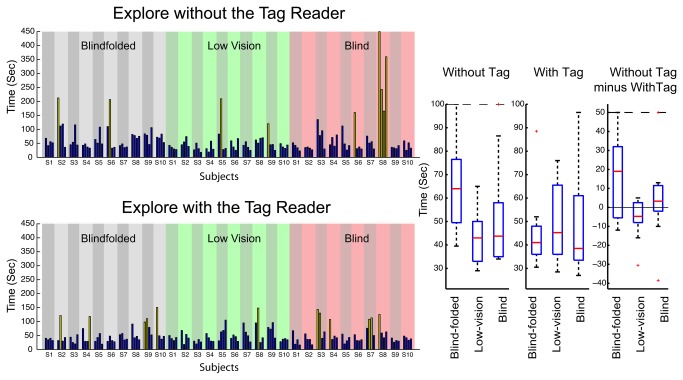
Exploration Performance. The bar plots show trial-by-trial times-to-completion for individual subjects in the three groups when they used the tag reader (lower panels) or did not use the tag reader (upper panels). Yellow bars designate outliers. Two box plots at the right show the distributions of the times-to-completion for the three groups of subjects in terms of the median times (red lines), 25% and 75% quartile limits (blue boxes), the maximum and minimum values (whiskers), and outliers (red crosses) for the three groups. Upper and lower dash lines, when present, mark the preset range limits if any outliers are outside the limits. Separate box plots show times for testing with and without the tag reader. A third box plot shows the within-subjects difference in times-to-completion with and without the tag reader.

The bar plots in Figure 7 illustrate trial-by-trial times for the three groups, lower panels when they used the tag reader and upper panels when they performed the task without the tag reader. The four bars for each subject represent the time in seconds to get to the target room in individual trials. The yellow bars designate outliers. These were trials where subjects took a long time to find the target room because they initially went the wrong way, overshot the room, or had some other problem.

All subjects and groups were able to successfully complete the tasks, with median group times ranging from 64 s (blindfolded group without tag reader) to 38 s (blind group with the tag reader). The times-to-completion decreased notably (i.e., faster performance) when using the tag reader for the blindfolded group (median within-subjects difference of 19 s). The tag reader made little difference in time-to-completion for the blind and low-vision groups (median within-subjects difference was 3 s (faster) for the blind group and -5 s (slower) for the low-vision group). Typically, the low-vision subjects used the Building Navigator software to determine the direction and distance of the target room, and then walked to it, sometimes counting the number of office doors passed en route as landmarks.

The sighted control group who performed the task without any technology had a mean time of 9.2 s. The three groups who used DSS for exploration took substantially longer than the sighted control group. A major portion of the extra time was devoted to using the DSS interface to find information about the direction and distance to the target room before beginning to walk. Although we did not formally subdivide the time-to-completion into “interface time” and “walking time,” we informally estimate that about half of the time was devoted to interface manipulation. It is also possible that our visually-impaired and blindfolded subjects walked more slowly, on average, than our sighted controls

We conclude from Experiment 2 that the DSS technology is useful in locating nearby points of interest in a corridor and walking to them. For this purpose, the digital-map functionality may be sufficient for low-vision and experienced blind users. The tag reader and digital tags facilitate performance for subjects who are inexperienced with non-visual mobility, suggesting that the reader technology may be most valuable for blind users who are less proficient in navigation. The ergonomic demands of the DSS technology mean that non-visual exploration is slower than corresponding exploration by sighted pedestrians.

### Experiment 3. Route Finding

**Figure 8:**
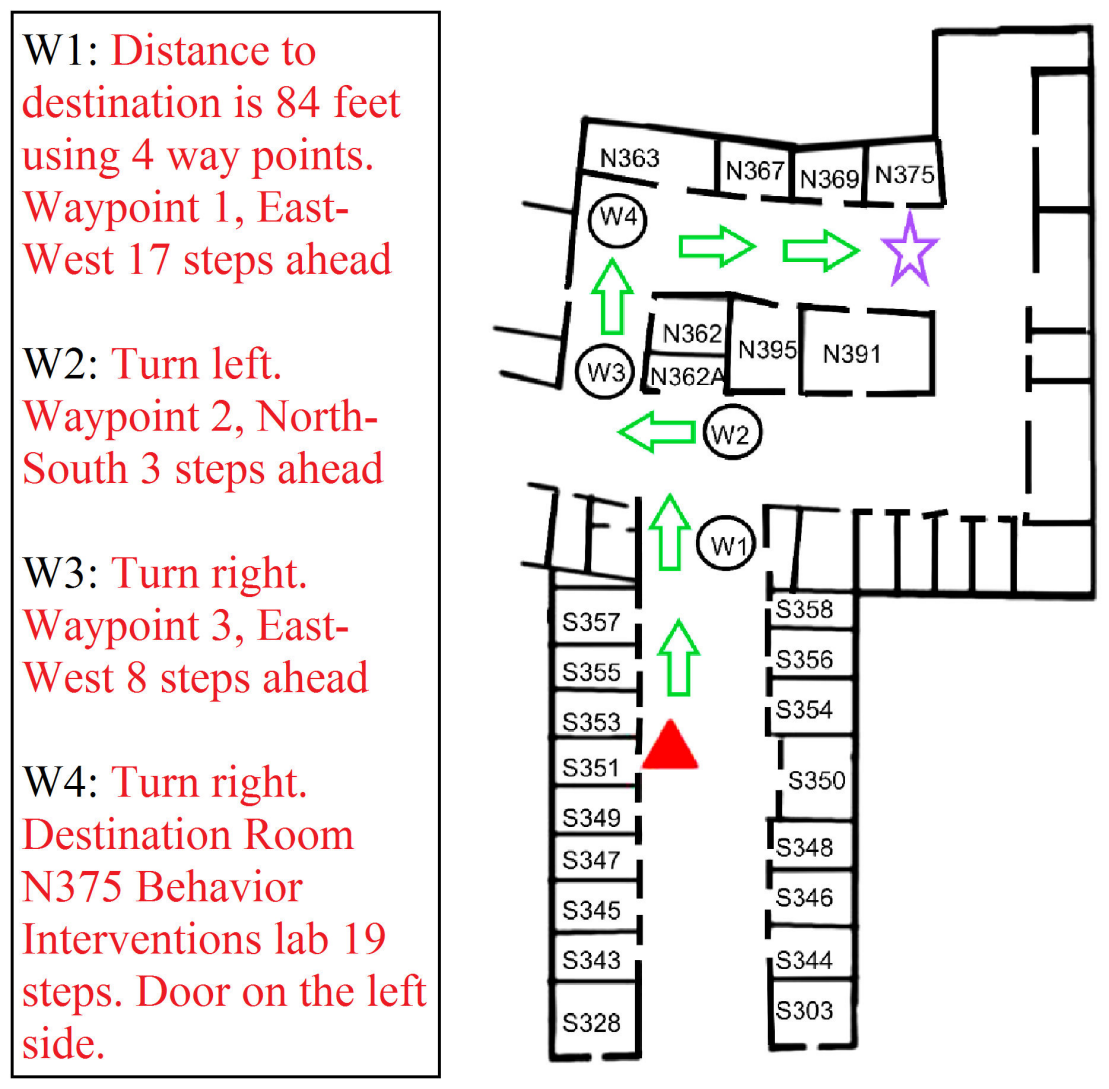
Building Navigator Route Finding Mode. A subject, located at the red triangle, wishes to travel to the target location marked by the purple star. Waypoints are indicated by circles. Note that “East-West” and “North-South” in the verbal descriptions refer to corridors. See the text for a description of the functionality of the route-finding mode.

Figure 8 illustrates the DSS Route Finding mode.

Similar to the map in Figure 6, the red triangle indicates a pedestrian’s starting location. The pedestrian’s destination is room N375, marked by the purple star. Building Navigator computes a route from the pedestrian’s current location to the destination. The route is represented as a series of waypoints. The interface presents the user with a list containing instructions for moving from waypoint to waypoint. Figure 8 shows the four waypoints for reaching room N375 and the table shows the corresponding list of verbal instructions. Typically, subjects listen to the instructions for reaching Waypoint 1, and then proceed. When they feel confident they have reached Waypoint 1, they listen to the instructions for Waypoint 2 and proceed, and so on. When the tag reader is available, they can monitor the rooms en route. DSS reports “On Route” or “Off Route” when a digital sign is read in Route Finding mode.

If a user gets off route and becomes lost, he or she can enter a new starting location by identifying whatever room might be nearby, and then compute a new route to the destination.

We tested the Route Finding mode by placing subjects at a starting location and asked them to use DSS to find a destination room on the same floor.

Each subject followed three routes each with and without the tag reader in counterbalanced order. The sighted control group simply used vision to find the target room and walk to it; they did not use DSS technology but had to rely on building signage including posted room numbers and maps. The average length of the routes was 214 ft and the routes had either four or five waypoints. We timed how long it took subjects to travel from the starting point to the target room.

**Figure 9:**
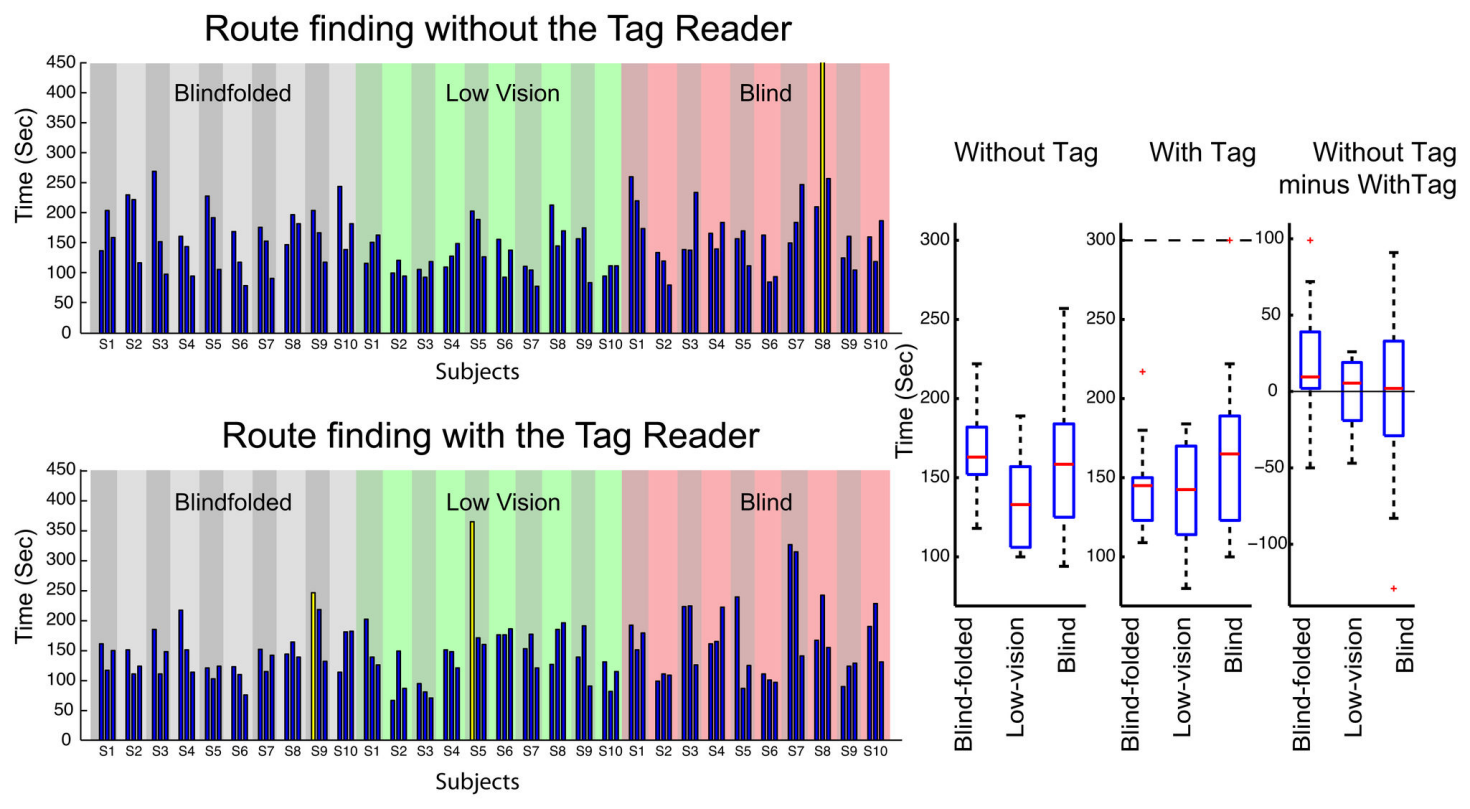
Route-Finding Performance. The bar plots show trial-by-trial times for individual subjects in the three groups when they used the tag reader (lower panels) or did not use the tag reader (upper panels). Yellow bars designate outliers. Two box plots at the right show the distributions of the times-to-completion for the three groups of subjects in terms of the median times (red lines), 25% and 75% quartile limits (blue boxes), the maximum and minimum values (whiskers), and outliers (red crosses) for the three groups. Upper and lower dash lines, when present, mark the preset range limits if any outliers are outside the limits. Separate box plots show times for testing with and without the tag reader. A third box plot shows the within-subjects difference in times-to-completion with and without the tag reader.

Figure 9 shows trial-by-trial times and summary box plots for the three groups in the route-finding experiment. Once again, all subjects and groups were able to successfully complete the tasks. Median group times ranged from 163 s (blindfolded group without tag reader) to 133 s (low-vision group without the tag reader). These times were more than twice the mean time of 61.9 s for the sighted controls who completed the route-finding tasks without use of the DSS technology. Once again, a substantial portion of this time was devoted to listening to the DSS interface and interpreting the verbal waypoint instructions. Despite the longer times, the subjects were successful in using DSS to accomplish a challenging wayfinding task.

Similar to the results in the Explore mode, the blindfolded group showed the greatest benefit of the tag reader (median within-subjects difference of 10 s). The median times for the blind and low-vision groups were similar for the two conditions. It should be noted, however, that a major benefit of the tag reader in route finding occurs when subjects go off route and need to recompute the route to the destination. For our subjects and routes, this scenario rarely occurred. When it did, the experimenter returned the subject to an on-route point and did not require them to recompute the route.

An informal observation emerged regarding the five subjects who used dog guides (S3, S4, S5, S6 and S8 in the blind group in Figure 9). The waypoints typically occurred at intersections. The dogs were effective in locating intersections and pausing at them.

We conclude from Experiment 3 that the DSS technology is useful in following routes to a remote destination in a relatively complex indoor corridor layout. It appears that digital-map functionality by itself may often be sufficient for route finding by experienced visually-impaired pedestrians. The sign reader and digital signs may be more helpful for subjects with less experience navigating without visual input. Once again, the ergonomic demands of the DSS technology mean that non-visual route finding is slower than corresponding route finding by sighted pedestrians.

### Questionnaire Results

Following the three experiments, a questionnaire was administered to obtain feedback from our blind and low-vision subjects. Our subjects were enthusiastic about the goals of the project and the potential of the DSS technology. All 20 of our blind and low-vision subjects answered “Yes” to two Yes/No questions regarding the need for adaptive technology for indoor navigation:

- Q1. Do you think there is a need for an indoor electronic travel aid for visually impaired people?
- Q2. If properly designed and affordable, would you use such an indoor travel aid?

Eight additional questions asked the subjects to rate DSS on a five-point scale from 1 (best) to 5 (worst). Table 1 lists the questions along with the means and standard deviations of the ratings.

**Table 1:** Questionnaire Data.

**Questions**	**Blind N = 10 Mean (s.d.)**	**Low Vision N = 10 Mean (s.d.)**
**Instruction: We showed you three functions of the travel aid we’re designing. Please rate the importance of these three functions for any travel aid, using a scale from 1 (very important) to 5 (not important)**		
**Q3. Browsing (naming features such as door numbers as you move through a space)**	1.40 (0.97)	2.20 (1.32)
**Q4. Exploring the Immediate Environment (listing direction and distance to nearby features, such as rooms)**	1.80 (0.79)	1.90 (0.99)
**Q5. Route Finding (creating a list of waypoints from your current location to a destination)**	1.30 (0.48)	1.50 (0.71)
**Instruction: In our study, we tested you with and without the Sign Reader in the Explore experiment and the Route Finding experiment. Please rate the effectiveness of the technology with and without the sign reader, using the 5-point scale from 1 (very effective) to 5 (not effective).**		
**Q6. Explore WITHOUT the Sign reader (finding a nearby room)**	2.10 (0.99)	2.10 (1.20)
**Q7. Explore WITH the Sign reader (finding a nearby room)**	1.50 (0.97)	1.90 (1.37)
**Q8. Route finding WITHOUT the sign reader**	2.30 (1.16)	1.60 (0.97)
**Q9. Route finding WITH the sign reader**	1.40 (0.97)	1.90 (1.37)
**Instruction: From what you have seen today, and assuming our DSS system could be packaged as a cell phone app or other small handheld device:**		
**Q10. please rate its potential effectiveness as an indoor travel aid from 1 (very effective) to 5 (not effective)**	1.33 (0.71)	1.20 (0.42)

Both blind and low-vision subjects rated the three DSS modes—Tag Browsing, Exploration and Route Finding—as important (Q3 – Q5). The blind subjects assigned greater importance to them than the low-vision subjects. Both groups considered the Route finding mode as most important.

Both groups indicated that DSS could be useful for exploration and route finding with or without the tag reader (Q6 – Q9). This result emphasizes that access to a talking digital map can be useful even in the absence of location sensing.

Finally, both groups rated the overall potential of DSS as very high (Q10).

The subjects were also given an opportunity to make open-ended narrative comments. Most reported liking the familiar phone keypad interface, assignment of keys to functions, the user-specific settings for the interface, and the availability of hazard and other semantic information. Concerns were expressed about the size of the tag reader, the need for two devices (tag reader and cellphone or tablet), and the requirement to properly orient the tag reader. Some users noted that they walked so quickly that the synthetic-speech output lagged behind their current position, causing temporary confusion.

## General Discussion

In the Introduction, we identified three key components for adaptive technology for indoor navigation—location sensing, an appropriate digital map of the space, and a suitable human-machine interface. How well does DSS meet these requirements?

The digital tags and tag reader provide a mechanism for location sensing. The tags are cheap to produce and easy to install. They are relatively unobtrusive and robust. Over a period of two years of use, only a small fraction of the installed tags on several floors of two buildings were damaged or disappeared.

Experiment 1 demonstrated that the tags can be reliably detected and read during normal mobility, provided that the tags are mounted at a consistent height within the operating distance of the camera, and the user has a rough idea of their direction in space. Finding and reading the tags non-visually is more challenging in an open space, such as a lobby, where the locations of the tags are less predictable. While it has become relatively commonplace for sighted people to use a camera phone to link to information through a QR code or other 2-D bar codes, our results indicate that information can be acquired from digital signs by visually-impaired pedestrians during active mobility.

A limitation of DSS is that location sensing requires active search with a camera for discrete position markers. Accuracy is limited by the number and distribution of digital signs in a layout and by the ability of the user to image the signs. By comparison, GPS typically provides continuous spatial updating in outdoor environments. Indoor systems for location sensing, such as Wi-Fi or Bluetooth triangulation, could possibly provide continuous monitoring indoors, but would likely require dedicated and strategically placed transmitters. One advantage of DSS over these technologies is that it provides immediate information about the user’s orientation and is not reliant on sampling across time to infer direction of movement.

While software methods abound for portraying building maps, we quickly learned to appreciate the challenge of creating a map data structure amenable to verbal descriptions. We designed our own digital-map software for inclusion in DSS. The maps contain two essential types of information for indoor mobility—spatial and semantic. The spatial information encodes the navigable geometry of the layout, especially features such as rooms, corridors and lobbies. The semantic information associates text descriptors with the geometrical features such as names on room doors or the contents of offices. While the geometrical (architectural) features of a building are usually stable over time, semantic information such as the names on doors is less stable. Long-term use of DSS in a building would require periodic updating of the digital maps, especially their semantic content.

We note that the technology for location sensing (digital signs and tag reader) provided relatively small benefits in performance in Exp. 2 (Exploration) and Exp. 3 (Route Following). For these tasks, the digital map and speech interface were useful on their own. These results demonstrate that a talking digital map of an indoor space can sometimes be useful without technology for location sensing. Nevertheless, the location sensing capability of the system is valuable in several contexts such as in monitoring points of interest during mobility (as in Exp. 1 on Tag Browsing), or in establishing current position in the Exploration mode, or in recomputing routes if/when lost in the Routing mode.

The DSS interface uses synthetic speech for output, reported to be preferable to auditory icons or tactile output [18]. While we have not implemented braille output, it would be straightforward to route the interface’s verbal messages to an electronic braille display. The output messages consist of simple descriptions of local building geometry, and semantic information about the space in several levels of verbosity. The success of our subjects in the exploration and route-finding experiments confirm that the simple geometrical descriptions are sufficient for successful building navigation. Previous research has shown that simple verbal descriptions of local building geometry can support the formation of cognitive maps of building layouts [23-25].

Our experiments did not test the substantial semantic capabilities of DSS, i.e., the utility of the verbose and hazard descriptions illustrated in Figure 6. Nevertheless, we believe these features were appealing to our subjects and constitute a functionally important component of the system.

We now address the four goals listed earlier for the human performance testing:

First, we wished to test the reliability and functionality of the integrated system of digital tags, tag reader, digital maps, and human interface. Following several iterations of software and hardware development, the integrated system worked well during the performance described in this paper. We conclude that the goal of integrating the hardware and software components of DSS was achieved.

Second, the primary goal of performance testing was to determine if participants could use the software and hardware to accomplish the intended functions of the system. The high reliability (> 90%) of tag identification during active mobility (Exp. 1), and the success of subjects in accomplishing the exploration and route-finding tasks (Experiments 2 and 3) confirm that our subjects could use DSS for its intended functions. In brief, our subjects could independently use DSS to browse room numbers as they traversed a corridor, find a target room in a corridor, and follow a route to a destination room at a remote part of the floor. In a building without tactile signage, all of these activities would normally require assistance from a sighted person.

A third goal was to assess the accuracy and speed of the participants’ independent use of DSS for indoor navigation. Our subjects were accurate in using DSS to accomplish the exploration and route-finding tasks, but it took them substantially more time than the sighted controls. Much of the extra time was taken in accessing and listening to the speech interface. It is possible that improved interface design, or more practice with the system by subjects would reduce interface time.

Finally, we evaluated performance differences between our three groups of subjects. The blind and low-vision subjects were selected on the basis of self-reported mobility skills. The blindfolded sighted group may be representative of individuals with little experience with non-visual mobility. Differences between the groups were hard to discern because of the substantial individual variability. The blindfolded sighted group appeared to show the greatest benefit from the tag reader, perhaps indicating that feedback on current position is especially helpful for inexperienced blind travelers.

The questionnaire results indicated that both blind and low-vision subjects were positive about the potential of the technology, with the blind subjects finding the specific features somewhat more important than the low-vision subjects.

The consensus of our subjects was that the prototype hardware, while functional in its current form, has two limitations. First, the current tag reader is too bulky for convenient use while managing a cane, dog guide or carrying a purse or other items; the tag reader is about the size of a 3-cell heavy-duty flashlight. Second, a practical system should integrate the tag reader and mobile device into a single package. Several of our subjects use iPhones with VoiceOver software and expressed the wish that DSS could be packaged as an iPhone app using the iPhone’s camera.
